# The Lack of Standardized Outcomes for Surgical Salvage of HPV-Positive Recurrent Oropharyngeal Squamous Cell Carcinoma: A Systematic Scoping Review

**DOI:** 10.3390/cancers15102832

**Published:** 2023-05-19

**Authors:** April N. Taniguchi, Sarah R. Sutton, Shaun A. Nguyen, Alexandra E. Kejner, William G. Albergotti

**Affiliations:** 1 Department of Otolaryngology-Head and Neck Surgery, Medical University of South Carolina, Charleston, SC 29425, USA; 2 College of Medicine, University of Central Florida, Orlando, FL 32827, USA; 3 School of Medicine, University of Nevada, Reno, NV 89557, USA

**Keywords:** oropharyngeal squamous cell carcinoma (OPSCC), human papillomavirus (HPV), survival, salvage surgery

## Abstract

**Simple Summary:**

Despite salvage surgery being a key treatment option for recurrent oropharyngeal squamous cell carcinoma, it is unclear how human papillomavirus (HPV) status affects survival rates. The aim of this study was to evaluate how salvage surgery impacts patient survival in the setting of HPV oropharyngeal cancer that recurred within the head and neck region. This scoping review of 32 studies found that only 18.8% of these articles evaluated survival data specific to salvage surgery patients, and 4 studies inconsistently reported survival endpoints, such as overall survival. These findings suggest that future studies can improve upon survival reporting to guide clinical management decisions for HPV-positive recurrent oropharyngeal cancer.

**Abstract:**

Although HPV status is known to provide an improved prognosis in initial treatments of HPV-positive oropharyngeal squamous cell carcinoma (OPSCC), it is unclear how it affects patients who receive salvage surgery (SS), which has historically poor survival rates. The purpose of this study was to evaluate the role of SS for patients with locoregional recurrence (LRR) of HPV-positive OPSCC and its impact survival rates. We conducted a scoping review of literature through October 2022 and included 995 individuals. Survival endpoints, such as overall survival (OS), Kaplan–Meier curves, and median post-recurrence survival, were analyzed in addition to demographics. Of all studies, 18.8% (6/32) reported any survival data for SS patients, with the most prevalent reporting 2- and 5-year OS in two studies. Median post-recurrence survival was not reported for SS. These findings reveal the limited and unpredictable reporting of survival-specific data on SS for HPV-positive OPSCC. With limited survival assessment, it is difficult to assess the potential advantages and disadvantages of this therapy to guide clinical decision-making.

## 1. Introduction

The prevalence of human-papillomavirus (HPV)-positive oropharyngeal squamous cell carcinoma (OPSCC) continues to increase in the United States and internationally [1,2]. Patients with HPV-positive OPSCC tend to be younger and healthier than their HPV-negative counterparts, and despite the improved prognosis of HPV-associated cancers, recurrences still occur locoregionally and metastatically at rates of 17.3% and 6.5%, respectively [3]. Most primary HPV-positive OPSCC cases, however, are successfully treated with surgery or radiation-based therapy [4]. For those that recur, particularly after radiation-based therapy, salvage surgery (SS) is often considered the best-remaining curative treatment option [5,6]. Despite being the best treatment option, SS carries significant morbidity and has been previously reported to have poor survival outcomes for OPSCC in general, but it is not known how the prognosis differs between HPV-positive and HPV-negative cancers [7].

Previous studies have either failed to address the role of HPV status in recurrent OPSCC or acknowledged the lack of strong evidence available [5,6,8,9]. It has been labelled as a potential positive prognosticator in SS [10], yet the current literature is ambiguous about the role of HPV status on survival outcomes of SS, with some reports of positive effects [11,12] and others with no impact [13,14]. Based on these findings, our goal was to investigate the relationship between HPV-positive OPSCC and SS outcomes.

The primary aim of this study was to evaluate the impact of SS treatment on overall survival, disease-specific survival, progression-free survival, and recurrence-free survival outcomes for patients with HPV-positive OPSCC and to secondarily assess an association between patient characteristics and survival outcomes. Upon initial evaluation of the literature, it was discovered that cancer survival rates were inconsistently reported. Therefore, a scoping review was conducted. The objective of this review is to summarize the reporting of survival outcomes for adults (≥18 years) with HPV-positive OPSCC who received SS for recurrent disease and highlight the clinical importance of the current limitations in the literature.

## 2. Materials and Methods

This systematic scoping review was conducted in accordance with Preferred Reporting Items for Systematic Reviews and Meta-Analyses Extension for Scoping Reviews (PRISMA-ScR) guidelines [15] and followed the five-stage framework outlined by Arksey and O’Malley [16]. A preliminary search of PubMed and the Cochrane Database of Systematic Reviews was conducted, and no current scoping reviews on the topic were identified.

### 2.1. Identify the Research Question

This review sought to investigate the reporting of survival outcomes for adults (≥18 years) with HPV-positive OPSCC who received SS for recurrent disease. Additionally, we wanted to highlight the clinical importance of the current limitations in the literature.

### 2.2. Identify Relevant Literature

With the help of an academic librarian, a search strategy was developed and was executed by the two primary authors, A.N.T. and S.R.S. A systematic search was conducted with the PubMed (US National Library of Medicine, National Institutes of Health), SCOPUS (Elsevier), and CINAHL (EBSC) databases from the inception through 10 October 2022, using keywords related to oropharyngeal neoplasms, recurrent disease, HPV, and salvage therapy. The PubMed search was adapted for the other two databases by replacing MeSH terms with the appropriate subject headings, when possible, and maintaining similar keywords. This strategy is detailed in the (Supplement Table S1). Additional studies were selected after screening the reference list of all included sources of evidence. All articles from the search were exported into Covidence (Veritas Health Innovation Ltd., Melbourne, Australia), the review management software, for screening. All identified citations were collated and uploaded into EndNote X20/2021 (Clarivate Analytics, Philadelphia, PA, USA).

### 2.3. Study Selection

This review aimed to identify all published reports relevant to adults (≥18 years) with HPV-positive OPSCC. The population of interest was surgically salvaged for recurrent disease. This scoping review considered randomized controlled trials, non-randomized controlled trials, prospective and retrospective cohort studies, prospective and retrospective chart reviews, case–control studies, and case series studies. Review articles were assessed but not included in the reporting of quantitative data to avoid redundancy. Other exclusion criteria were non-human studies, non-English language, study protocols, and incomplete or inaccessible articles. There was no limitation for the time range of included publications.

#### Search Process

**Figure 1:**
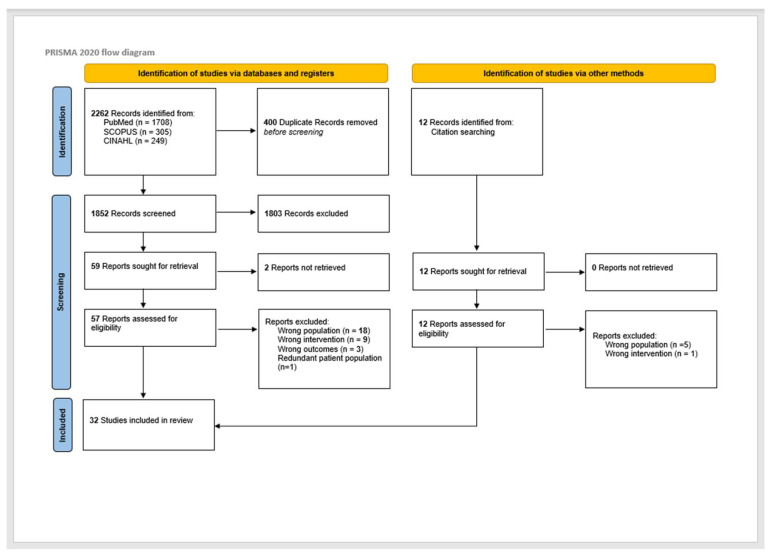
PRISMA diagram for study selection. ([17] Page MJ, McKenzie JE, Bossuyt PM, Boutron I, Hoffmann TC, Mulrow CD, et al. The PRISMA 2020 statement: an updated guideline for reporting systematic reviews. *BMJ* **2021**, *372*, n71. doi:10.1136/bmj.n71).

This review was conducted in compliance with PRISMA-ScR scoping review methods [15]. The initial search yielded 2262 reports. There were 400 duplicate reports. After duplicates were removed, 1852 titles and abstracts were screened by two independent reviewers (A.N.T. and S.R.S.) for assessment against the inclusion criteria, and conflicts were resolved by discussion. A total of 59 records were assessed for eligibility, with 31 reports excluded at the full-text stage. Eighteen reports had the wrong population. There were nine reports with the wrong intervention. Three reports were due to wrong outcomes, and one was due to redundant patient population. Thirty-two (26 from databases and registers and 6 from citation searching) full-text screenings of selected citations were done independently by the same reviewers (A.N.T. and S.R.S.), and any disagreements were resolved through discussion. The results of the study inclusion process are presented in a PRISMA-ScR flow diagram (Figure 1).

### 2.4. Charting the Data

#### 2.4.1. Data Extraction

Data extraction from the 32 included reports was first pilot tested, where both reviewers (A.N.T. and S.R.S.) independently extracted from ten articles, with over 90% agreement. All discrepancies were discussed, and adaptations were made to the extraction template. One author (A.N.T.) extracted the remaining reports. The data extracted from reports included author; year of publication; study design; aims of the study; key findings relevant to scoping review; patient demographics, such as age, gender, race, and risk factors; OPSCC characteristics; and survival parameters, which included overall survival, post-recurrence survival, cancer-specific survival, disease-free survival, progression-free survival, and Kaplan–Meier (K-M) survival curves.

#### 2.4.2. Level of Evidence and Risk of Bias

All the included reports were critically appraised to assess the level of evidence using the Oxford Center for Evidence-Based Medicine criteria [18]. The risk of bias was assessed according to the Cochrane Handbook for Systematic Reviews of Interventions (version 6.0.22) [19]. The level of evidence and the risk of bias were assessed by two reviewers (A.N.T. and S.R.S.). A pilot assessment on three reports was first performed to check for consistency of the assessment, and then, independent risk assessments were performed on the remaining studies. Conflicts related to the level of evidence for each article were first resolved by a discussion between the two reviewers (A.N.T. and S.R.S.) and then by a third reviewer (S.A.N.) when necessary. The Risk of Bias In Non-randomized Studies-of Interventions (ROBBINS-I) tool was used to evaluate the non-randomized study designs. Risk-of-bias items included the following: bias due to confounding, bias in selection of participants of the study, bias in classification of interventions, bias due to deviations from intended interventions, bias due to missing data, bias in measurement of outcomes, and bias in selection of the reported results. The risk of bias for each category is graded as low risk, high risk, or unclear risk.

### 2.5. Collating, Summarizing, and Reporting Results

Review results are presented through descriptive statistics (frequency (%), mean/median, and range/95% confidence interval (CI)) of included studies and a narrative summary of findings. Implications of the analysis are then discussed.

### 2.6. Definitions

The goal of this review was to evaluate commonly reported survival outcomes, such as overall survival (OS), progression-free survival (PFS), disease-specific survival (DSS), disease-free survival (DFS), and relapse-free survival (RFS).

OS is the percentage of patients that are still alive for a specified time after receiving a diagnosis or treatment. PFS is the length of time that a patient lives with a disease that does not worsen. DSS rate is the percentage of patients who have not died from a defined disease in a certain period. DFS is the length of time that a patient survives without signs or symptoms of his/her disease. DFS and PFS are markers that can determine the efficacy of a new treatment. Definitions are derived from the National Cancer Institute [20]. Additional survival data assessed was depicted graphically with K-M curves. Median values of post-recurrence survival were reported as the duration of time from initial detection of the recurrent disease until death or the end of the study [21].

In this study, patients were defined as having recurrent disease if the included studies reported their disease as recurrent or persistent. Patients in six of the included studies were labelled with persistent cancer seemingly based on the discovery of disease within <6 months of treatment. We did not view this distinction to warrant a separate category, so all patients documented with persistent or recurrent disease were grouped as recurrent. Recurrence refers to locoregional recurrence (LRR) and does not include distant metastasis. Time to recurrence was not routinely reported as a predictive variable of interest in the included studies.

SS, as defined in this study, was any surgical approach utilized for recurrent HPV-positive OPSCC. There was considerable variation in how SS was defined by the included studies, and some did not explicitly indicate which surgical approach was utilized. Other studies defined SS with the following methods: primary tumor resection with or without neck dissection (ND), ND, surgery with flap reconstruction, transoral robotic surgery (TORS), transoral laser microsurgery (TLM), open approach, total laryngectomy with or without glossectomy, mandibulectomy, mandibulotomy, and tonsillectomy.

### 2.7. Statistical Analysis

Meta-analysis of single means (mean, N for each study, and standard deviation) for age was performed by Comprehensive Meta-Analysis (version 3) (Biostat Inc., Englewood, NJ, USA). Meta-analysis of proportions (gender, race, smoking status, etc.) was performed using MedCalc 20.110 (MedCalc Software, Ostend, Belgium) and was expressed as a percentage with 95% confidence intervals. Each measure was weighted according to the number of patients affected. The weighted-summary proportion was calculated by the Freeman–Tukey transformation [22]. Heterogeneity among studies was assessed using χ^2^ and I^2^ statistics. If there was high heterogeneity (I^2^ > 50%), the random-effects model was used; if heterogeneity was low, then a fixed-effects model was considered allowable [23].

## 3. Results

### 3.1. Publication Characteristics

The search for surgical salvage in patients with HPV-positive OPSCC encompassed 2264 articles, of which 26 were included [11,12,14,24,25,26,27,28,29,30,31,32,33,34,35,36,37,38,39,40,41,42,43,44,45]. An additional 6 articles were incorporated by hand searching the reference lists of eligible studies, thus yielding 32 articles [46,47,48,49,50,51]. As illustrated in Figure 1, the 32 included studies were published between 2013 and 2022 and largely categorized as Level 4 evidence. Most of the articles originated from the United States and were retrospective chart reviews (Supplemental Table S2) [12,27,30,32,34,35,36,37,38,39,40,41,42,44,45,46,47,48,49,50]. Appraisal of studies reveals an appropriately low risk of bias, with potential sources related to bias in selection of participants and bias due to confounding.

### 3.2. Patient Attributes

Most of the studies presented detailed demographics for subjects with a primary diagnosis of HPV-positive OPSCC [25,26,33,34,35,36,38,39,40,42,44,45,48,49,50,51], while others assessed those with recurrence only [11,12,24,27,28,30,32,41,46]. In six studies, either initial or additional demographic information specific to surgically salvaged patients was presented [13,14,29,31,43,47]. A total of 4444 HPV-positive OPSCC patients were included in this review. Of these, a subset of 995 patients had LRR, of whom 587 patients were treated with surgical salvage (Table 1). The mean age was 58.7 years with a standard error of 1.3 years, and the overall proportion of men and smokers was 87.9% [95% CI 81.5–93.1] and 51.6% [95% CI 14.0–88.1], respectively. The proportion of individuals of white race was 88.2% [95% CI 83.5–92.2]. The median time to recurrence was between 6 and 24 months for most (425/587; 72.4%) SS patients but was less than 6 months or unknown for the remaining patients.

### 3.3. Survival Outcomes

**Figure 2:**
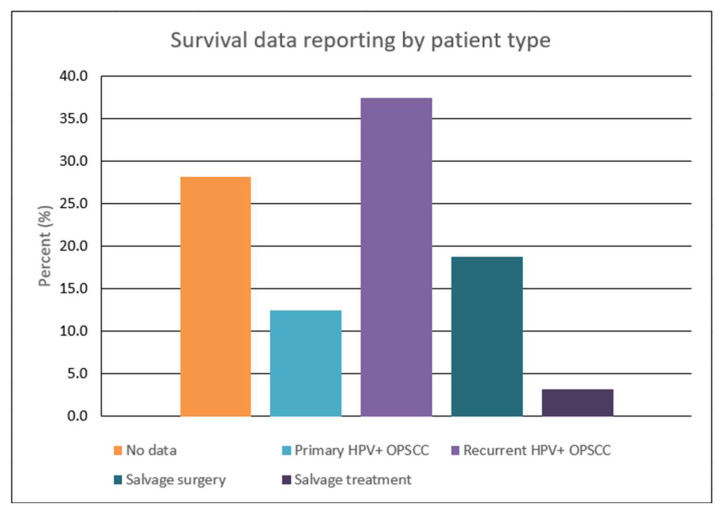
Graphical representation of survival reporting stratified by patient population.

Analysis of the 32 included articles demonstrated considerable variability in survival assessment for our study’s population of interest (Figure 2). A noteworthy finding was the lack of any survival data in 28.1% of studies. Only 18.8% contained survival outcomes (OS, DSS, and PFS) or K-M curves following SS. Survival evaluations were conducted for various groups, ranging from those with a primary diagnosis of HPV-positive OPSCC to those who received salvage treatments. Furthermore, the studies were not standardized in the type of assessment tools used and their respective survival timelines. Common methods for reporting these findings were survival parameters (e.g., OS and DFS), K-M curves, and median post-recurrence survival durations.

Unique to surgically salvaged patients, there was a paucity of evidence for survival endpoints, such as OS, DFS, and PFS. Twelve studies did not provide a numerical assessment of these variables, and the bulk of the remaining articles limited their evaluation to patients with a primary diagnosis of HPV-positive OPSCC or documented recurrence. A total of four articles calculated numerical values for survival endpoints (Table 2) [13,14,31,37]. However, these parameters were reported inconsistently, with two studies reporting 2-year OS [13,31] and two recording 5-year OS [13,14]. For 1-year OS, 2-year PFS, and 5-year RFS, these parameters were each published in one study [30,31,37]. Of the two studies that reported 2-year OS, the values were 91.3% and 51.0%, while the values for the two reporting 5-year OS were determined to be 43.0% and 27.0% [13,31].

**Figure 3:**
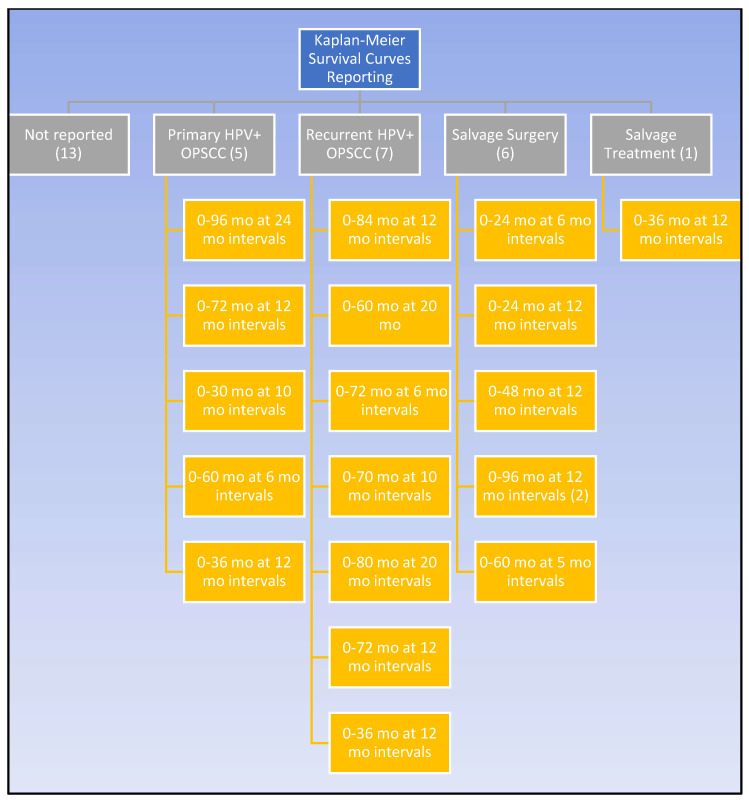
Kaplan–Meier reporting amongst the studies.

In addition to the numerical parameters, survival was graphically appraised through K-M curves. As exhibited by Figure 3, K-M survival curves were displayed in over half of the studies but with tremendous irregularity. The time frame and interval markers of the K-M curves differed greatly between studies. Like the survival outcomes, K-M curves assessed various patient groups, such as patients with primary HPV-positive OPSCC disease, patients with recurrent disease, and patients treated with any salvage treatment. SS patients specifically were limited to six articles. Of these six studies, only two shared the same time range and interval markers [11,12,13,14,30,31,37,52]. Median post-recurrence survival was reported in four studies, ranging from 13–151.2 months; however, this was not specific to SS patients [27,30,35,42,46].

## 4. Discussion

As a cancer treatment with curative potential, SS is a favorable option for patients with relapsed disease [10]. However, competing interests must be balanced when considering SS, such as the chance for improved survival versus surgical morbidity, which is pronounced with surgical salvage; this includes fibrosis, nerve injury, need for vascularized reconstruction, and wound dehiscence, all which can lead to swallowing dysfunction and potential tracheotomy dependence [7,9,53]. These risks must be balanced against survival benefits. Reported 3-year survival rates of SS for OPC range from 34–62%, but there is inconclusive evidence on SS in HPV-positive patients with recurrent OPSCC due to conflicting results [11,37]. This scoping review has comprehensively investigated subjects who were surgically salvaged for HPV-positive recurrent OPSCC and identified the inadequate reporting of survival outcomes.

Salvage surgery is inherently more dangerous and carries more complications than its primary surgery counterpart because of fibrotic tissue planes, advanced disease, and patients who are infirm and malnourished from prior treatments [7,53]. Optimal patient selection is thus paramount [8]. Prior studies have advocated for the use of the Charlson Age Comorbidity Index (CACI) as a prediction model for SS patients with head and neck squamous cell carcinoma (HNSCC) [10], which reveals the advantage of analyzing patient characteristics. With careful evaluation, Kim et al. concluded that medical comorbidity and age, primary T3 or T4 stage, and a disease-free interval of under 6 months were independent risk factors for death after SS of HNSCC [54]. These findings, if known for HPV-positive OPSCC SS patients, would be instructive in understanding and improving survival outcomes. It is recommended that reporting of SS patient demographics becomes standardized to optimize quality of life (QoL) and interpret which factors impact patient survival.

In order to optimize the survival outcomes of SS, it is important to recognize that not all subjects are good candidates for surgical intervention—both from a medical standpoint and from an anatomic standpoint [8]. Careful pre-operative assessment should be performed to determine the patients’ functional statuses and their propensity for clinical deterioration [10]. Of note, malnutrition is a major problem seen in patients with head and neck cancer, which is often attributed to both tumor location and the side effects of primary radiation and chemoradiation [55]. With symptoms such as odynophagia, mucositis, and xerostomia, patients can experience substantial swallowing dysfunction and become dependent on a percutaneous endoscopic gastrostomy (PEG) tube for nutritional support [55]. Evaluation of abilities such as swallowing is a crucial consideration for patients and surgeons alike, especially with the sequelae of primary treatment. Of the 587 SS patients described in the included studies, there was a limited assessment of this group’s demographics. This review found a modest six studies that reported attributes such as sex, age, race, smoking status, and primary tumor location [13,14,29,31,43,47]. Other traits, such as alcohol usage, blood glucose control, cardiovascular disease, renal disease, and pulmonary function, were not documented despite their considerable influence on healing and post-operative complications [8,10]. Although most of the SS patients had a median disease-free interval >6 months, there was considerable variability reported among the studies. Time to recurrence was assessed as a predictive variable in one study for all recurrent patients but in zero for patients managed with SS [27]. Time to recurrence has been cited as the single most important factor affecting survival of recurrent disease and may indicate tumor virulence [5]. As such, it would be optimal to stratify patients by the time to recurrence, as seen in the study by Zafereo et al., for the most accurate survival assessment [7].

In this scoping review of HPV-positive recurrent OPSCC SS patients, the literature demonstrated a scarcity of survival variables, with the most robust reporting from two studies that evaluated OS at 2 and 5 years (Table 2). This was unanticipated given the improved outcomes seen with SS of recurrent OPSCC, such as a 3-year OS of 74% compared to that of 11% with nonsurgical treatment [7]. The findings of this review highlight the opportunity for several improvements in survival analysis. In addition to more consistent reporting for HPV-positive patients, evaluation should include underreported variables, such as PFS and DFS, that are markers of treatment efficacy in clinical trials [20,56]. These markers of PFS and DFS were only present in one of the included studies as a 2-year PFS of 82.6% [31]. This sparse reporting reveals a considerable knowledge gap because most treatment failures or recurrences happen within 2 years, and measuring disease control at that point provides insight into treatment efficacy [5]. For instance, a meta-analysis by Goodwin discovered a surprisingly positive 2-year DFS of 51% for SS patients, including those with advanced recurrent disease [5]. These clinical outcomes are useful in guiding management decisions and should be further evaluated with respect to HPV-positive oncologic patients.

Despite their frequent use among the included studies, K-M curves had a limited benefit in expanding the knowledge of SS survival projections for HPV-positive recurrent OPSCC. Out of 32 studies, only 6 published K-M curves for surgically salvaged patients [11,12,13,14,30,31,37], and 2 of those had the same timeline [14,37]. Without standardized time intervals and durations, it is difficult and potentially misleading to compare K-M curves between different study populations. Without reliable comparisons, K-M curves cannot be used to determine whether patient characteristics impact the survival outcomes, and this hinders efforts to draw conclusions from a larger population. Additionally, there are caveats for all K-M curves. Even if two groups visually differ on a curve, they may fail to be significantly different [57]. The data’s reliability decreases with fewer patients, and curves with an unknown or high number of censored patients should be cautiously interpreted [57]. Considering how often K-M curves are reported as survival data, it is recommended that they are modeled after curves with larger groups and demarcated censored patients but, most importantly, interpreted in context with other measures [11,12,13,14,25,27,28,29,30,31,32,34,37,38,40,41,46,48,49,52].

As further evidence of reporting insufficiency, zero studies documented median post-recurrence survival (PRS) for surgically salvaged patients with recurrent HPV-positive OPSCC. Instead, four studies reported median PRS for those patients with any treatment modality [27,30,35,42]. The range from 13–151.2 months conveyed the potential lifespan after disease recurrence yet was uninformative about SS outcomes. Furthermore, PRS is calculated by the K-M method and can also be presented as yearly rates like other endpoints (OS and PFS) for SS [21,58]. In this format, PRS has been used to determine whether there was a survival benefit after surgical resection in recurrent esophageal cancer [21]. Although SS was not significantly superior to other treatments, this prior study has shown the benefit of using PRS rates to assess the treatment’s effect on patient survival for recurrent cancer [21].

To improve clinical decision making, the focus of this study was to emphasize the underreporting of survival data for the growing population of HPV-positive recurrent OPSCC SS patients. Survival endpoints, such as the 2-year PFS and DFS, would be a helpful tool when considering SS treatment for patients [20,56]. It is advantageous to present survival parameters numerically (e.g., 5-year OS of 50%) because they are more straightforward to interpret and do not require analysis of an entire K-M mortality curve [57]. It is important to improve transparency because cancer survival statistics can be difficult for some clinicians and patients to accurately interpret [59]. As nonsurgical therapies, such as checkpoint inhibition and other targeted therapies, evolve for the treatment of recurrent HPV-positive OPSCC, it will be helpful to have baseline SS survival data to compare against in the absence of direct trial comparison.

Given the distinct nature of HPV-positive and -negative oropharyngeal cancers, we recommend that future studies consistently record HPV status to distinguish between the two patient groups and not aggregate the data as one population. Furthermore, with a similar trend of limited survival reporting for HPV-negative patients, direct comparisons between surgically salvaged HPV-positive and -negative patients could reveal whether HPV status is a reliable prognostic factor.

With 75% of the articles being retrospective chart or cohort studies (Table S2), this review was limited by the quality and availability of the data. The study patients were grouped into categories of primary disease, recurrent disease, SS, and salvage treatments, as seen in Figure 2 and Figure 3. Our methodology included combining patients that had local and regional recurrence into LRR, as seen in previous literature [12,38]. Another limitation is the cautious interpretation of three studies that performed a secondary analysis on patients from clinical trials RTOG 0129 and/or 0522 with unique inclusion criteria but potentially overlapping subjects [11,24,31]. As with any tool, there are inherent limitations to survival measures. Even the most common and reliable measure of OS is not strictly limited to the cancer, and survival rates can be affected by causes of death besides cancer [59]. It is vital to have a shared decision-making approach that considers all the patient’s wishes in addition to survival.

## 5. Conclusions

It has been over 20 years since Goodwin concluded that SS was justified in recurrent UADT cancer [5], yet the literature on SS survival outcomes continues to produce insufficient results for recurrent HPV-positive OPSCC [11,37]. Less than 22% of studies provided any type of survival data for these patients. This missing data predictably hinders efforts to assess the patient characteristics of a successful salvage and to optimize preoperative selection. We recommend that future assessments of surgically salvaged recurrent HPV-positive OPSCC patients include the following: documenting pertinent medical traits or a prediction model like CACI; prioritizing the consistent and expanded reporting of survival measures, especially PFS and DFS; and depicting K-M curves with standardized time frames and interval markers. Reliable, comprehensive reporting of patient characteristics and survival outcomes will promote a better understanding of the advantages and disadvantages of this therapy.

## Figures and Tables

**Table 1:** Distribution of patients with recurrent disease and those requiring salvage surgery.

Study	LRR ^1^	SS ^2^	SS + adj ^3^
Bigelow 2022 [24]	51	21	0
Billfalk-Kelly 2019 [48]	11	4	0
Bledsoe 2013 [49]	3	3	0
Carey 2020 [25]	32	11	3
Chen 2017 [51]	3	3	0
Chen 2021 [26]	11	9	2
Christopherson 2021 [27]	69	32	17
Culié 2021 [28]	55	38	0
Daniels 2020 [29]	36	19	0
Dave 2017 [46]	5	1	1
DeFelice 2021 [30]	10	6	0
Fakhry 2014 [11]	57	29	0
Galloway 2016 [31]	199	69	0
Garden 2014 [47]	20	14	0
Guo 2015 [12]	51	46	0
Jackson 2017 [50]	26	5	8
Joseph 2016 [32]	43	33	0
Landin 2022 [33]	26	5	8
Lee 2018 [34]	7	2	0
Masroor 2019 [35]	5	3	0
Mueller 2021 [36]	17	17	0
Patel 2016 [37]	19	19	0
Patel 2022 [14]	99	99	0
Pipkorn 2019 [38]	21	1	11
Routman 2017 [39]	12	0	5
Ryan 2021 [40]	22	8	1
Sims 2017 [41]	19	7	0
Su 2020 [42]	26	0	8
Sweeny 2016 [13]	9	9	0
Williamson 2021 [43]	3	3	0
Wotman 2019 [44]	20	6	0
Yokota 2021 [45]	2	0	2

^1^ LRR = locoregional relapse, ^2^ SS = salvage surgery, ^3^ SS + adj = salvage surgery plus adjuvant treatment.

**Table 2:** Survival endpoint reporting for SS patients.

Study	Variable	Time Period	Value	95% CI
Galloway 2016 [31]	PFS	2 year	82.6	73.7–91.6
	OS	2 year	91.3	84.7–98.0
Patel 2016 [37]	RFS	5 year	21.0	3.0–39.0
Patel 2022 [14]	OS	5 year	43.0	
Sweeney 2016 [13]	OS	2 year	51.0	
	OS	5 year	27.0	

## Data Availability

All data is available in the previously published studies.

## Supplementary Materials

The following supporting information can be downloaded at https://www.mdpi.com/article/10.3390/cancers15102832/s1, Table S1: Search Strategy, Table S2: Table of study characteristics.

## Abbreviations

**Abbreviation**	**Definition**
HPV	Human papillomavirus
OPSCC	Oropharyngeal squamous cell carcinoma
SS	Salvage surgery
OS	overall survival
PRS	post-recurrence survival
CSS	cancer-specific survival
DFS	disease-free survival
PFS	progression-free survival
K-M	Kaplan–Meier
DSS	disease-specific survival
RFS	relapse-free survival
LRR	Locoregional recurrence
ND	Neck dissection
TORS	Transoral robotic surgery
TLM	Transoral laser microsurgery
CACI	Charlson Age Comorbidity Index
HNSCC	Head and neck squamous cell carcinoma
QoL	Quality of life
PEG	Percutaneous endoscopic gastrostomy
