# Supplementary material for: The Lack of Standardized Outcomes for Surgical Salvage of HPV-Positive Recurrent Oropharyngeal Squamous Cell Carcinoma: A Systematic Scoping Review
Source: Cancers (Basel). 2023 May 19;15(10):2832. doi: 10.3390/cancers15102832 (PMC10216204; doi:10.3390/cancers15102832)
Supplement: Supplementary file 1 [file cancers-15-02832-s001.zip › cancers-2362652-supplementary.pdf]

Supplemental Table S1: Search strategy

Search conducted on October 10, 2022

PubMed: ("Oropharyngeal Neoplasms"[Mesh] OR Oropharyngeal Neoplasm* OR "oropharyngeal cancer"[tiab] OR "OPSCC"[tiab] OR "oropharyngeal squamous cell carcinoma "[tiab] OR "oropharyngeal carcinoma"[tiab] OR "tonsillar squamous cell carcinoma"[tiab] OR "tongue squamous cell carcinoma"[tiab]) AND ("Locoregional Recurrence"[tiab] OR Relapse* OR "Recurrent"[tiab] OR "recurrence"[tiab] OR "distant"[tiab] OR "metastatic"[tiab] OR "metastases"[tiab] OR "survival"[tiab]) AND ("Oropharyngeal Neoplasms/virology"[MeSH] OR "Carcinoma, Squamous Cell/virology"[Mesh] OR "Carcinoma, Squamous Cell/pathology"[Mesh] OR "human papilloma virus"[tw] OR "Neoplasm Recurrence, Local/virology"[Mesh] OR "HPV"[tiab] OR Human Papillomavirus OR P16-positive OR P16 status OR P16 OR "Cyclin-Dependent Kinase Inhibitor p16/genetics"[Mesh] OR human papillomavirus-positive OR human papillomavirus-associated OR HPV-associated [tiab] OR HPV-positive[tiab] OR "Papillomavirus Infections/complications"[Mesh]) OR "Papillomavirus Infections/complications"[Majr] AND ("Salvage Therapy"[Mesh] OR "Salvage Therapy/methods"[MeSH] OR "Neoplasm Recurrence, Local/surgery"[MAJR] OR "Oropharyngeal Neoplasms/surgery"[MAJR] OR "Oropharyngeal Neoplasms/therapy"[Mesh] OR "Salvage Surgery "[tiab] OR "Surgical salvage "[tiab] OR "Neck dissection "[tiab] OR "Neck Dissection/methods"[Mesh] OR "TORS"[tiab] OR "Transoral surgery"[tiab] OR "Transoral Robotic Surgery "[tiab] OR "failure"[tiab])	English filtered: 1708
SCOPUS: (TITLE-ABS-KEY ((oropharyngeal AND neoplasm OR oropharyngeal AND cancer OR opscc OR oropharyngeal AND squamous AND cell AND carcinoma OR oropharyngeal AND carcinoma)) AND TITLE-ABS-KEY ((relapse OR recurrent OR recurrence OR distant OR metastatic OR metastases OR survival)) AND TITLE-ABS-KEY ((hpv OR p16 OR human AND papillomavirus OR virology)) AND TITLE-ABS-KEY ((salvage OR surgery OR neck AND dissection OR surgical AND salvage OR cancer AND surgery)))	English filtered: 305
Cinahl: (oropharyngeal neoplasm OR oropharyngeal cancer OR opscc OR oropharyngeal squamous cell carcinoma OR oropharyngeal carcinoma) AND (relapse OR recurrent OR recurrence OR distant OR metastatic OR metastases OR survival) AND (hpv OR p16 OR human papillomavirus OR virology) AND (salvage OR surgery OR neck dissection OR surgical salvage OR cancer surgery)	English filtered: 249

Supplemental Table S2: Table of study characteristics

*Other refers to the following countries ≤ 1 article: France, Australia, Sweden, Germany, Japan, International

	Percent (%)
United States	65.6
Canada	9.4
United Kingdom	6.2
Other*	18.8
Total	100
Retrospective Chart	62.5
Retrospective Cohort	12.5
Case Series	3.1
Database	6.3
Single arm clinical trial	3.1
Case control	3.1
Analysis of clinical trial	9.4
Total	100
